# Ionic Liquid–Electrode Interface at Saturation: To Crowd, or Not to Crowd?

**DOI:** 10.1021/acs.jpclett.5c03710

**Published:** 2026-02-16

**Authors:** Ba Long Nguyen, Eva Roos Nerut, Aleksandr Beditski, Nadezda Kongi, Vladislav Ivanistsev, Iuliia V. Voroshylova

**Affiliations:** † Institute of Chemistry, 428488 University of Tartu, Ravila 14a, 50411 Tartu, Estonia; ‡ Faculty of Science and Engineering, Åbo Akademi University, Aurum, Henriksgatan 2, 20500 Turku, Finland; ¶ Institute of Mathematics, 214141 University of Tartu, Narva mnt 18, 51009 Tartu, Estonia; § Department of Chemistry, 61769 University of Latvia, Jelgavas iela 1, LV-1004 Riga, Latvia; ∥ LAQV-REQUIMTE, Department of Chemistry and Biochemistry, Faculty of Sciences, 131674 University of Porto, 4169-007 Porto, Portugal

## Abstract

At electrified interfaces, ions from concentrated electrolytes are known to arrange into alternating layers near highly charged surfaces. Saturation of such layers leads to a power-law decay in capacitance–potential curves. Some researchers relate this power-law to the crowding of ions; however, in this letter, we demonstrate that it can also result from the phenomenon known as overscreening. To help researchers answer the “to crowd or not to crowd” question, by distinguishing between these two charging regimes and ionic layer saturation, we derive an asymptotic description and examine molecular dynamics simulations, in which overscreening consistently dominates, while crowding is observed only under unrealistically extreme conditions. This insight expands understanding of how saturation works in concentrated electrolytes. Moreover, recognizing which mechanism is at play—crowding or overscreening—allows more precise control of charging behavior in electrochemical devices, from actuators, batteries, and capacitors to desalination systems, electrolyzers, and fuel cells.

## Graphical abstract

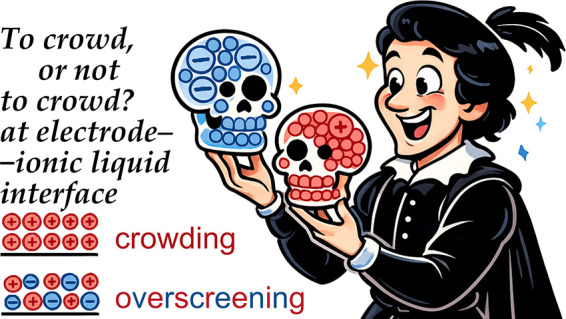

Although currently many theories of the electrical double layer (EDL) exist for concentrated electrolytes, such as ionic liquids (ILs),^1–7^ only a few of them and only in the most general way reproduce key structural features such as overscreening and crowding. These two phenomena represent distinct regimes of ion organization near charged interfaces: *overscreening* occurs when the contact layer overcompensates for the surface charge and subsequent layers gradually restore electroneutrality,^8,9^ whereas *crowding* arises when the contact layer cannot accommodate all the counterions required for full charge compensation.^4^ In many studies, it has not always been clear which regime of the “to crowd or not to crowd” question the interface is in. Some structural and behavioral aspects of these regimes are captured using computational (e.g., molecular dynamics, MD) simulations,^10^ experimental (e.g., atomic force microscopy, AFM) techniques,^11^ and theoretical (e.g., continuum theory) approaches.^4^

This letter presents a computational and theoretical perspective on the relationship between these charging regimes and the power-law dependence of the differential capacitance (*C*_d_) on the potential drop across the EDL (φ). Such a relation appears in the literature,^12–16^ yet, to our best knowledge, lacked explanation. For illustration, we use the *C*_d_(φ) data set presented in Figure 1a as obtained in MD simulations of a coarse-grained model of an IL–electrode interface. As shown in Figure 2, these simulations are standard in the field, and their methodological details are provided in Section SI1. Note that the power-law is evident from linearity in the log–log representation of *C*_d_(φ) in Figure 1b. Below, we explain peculiarities of that dependence and emphasize that the region highlighted with yellow in Figure 1a is commonly misidentified as crowding,^14,16–32^ while at closer inspection it becomes clear that what appears at first sight as crowding is in fact overscreening.

**Figure 1:**
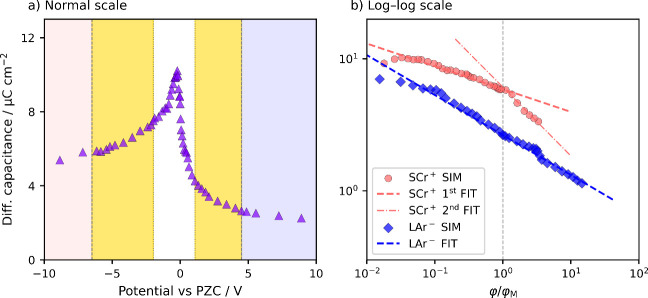
Differential capacitance dependence on the potential. (a) Data presented on the absolute scale. (b) Data presented on the relative scale relative to the PMC with dashed lines following eq 3 with α_LAr^–^_ = 0.70 and α_SCr^+^_ = 0.83 and 0.48 (above PMC).

**Figure 2:**
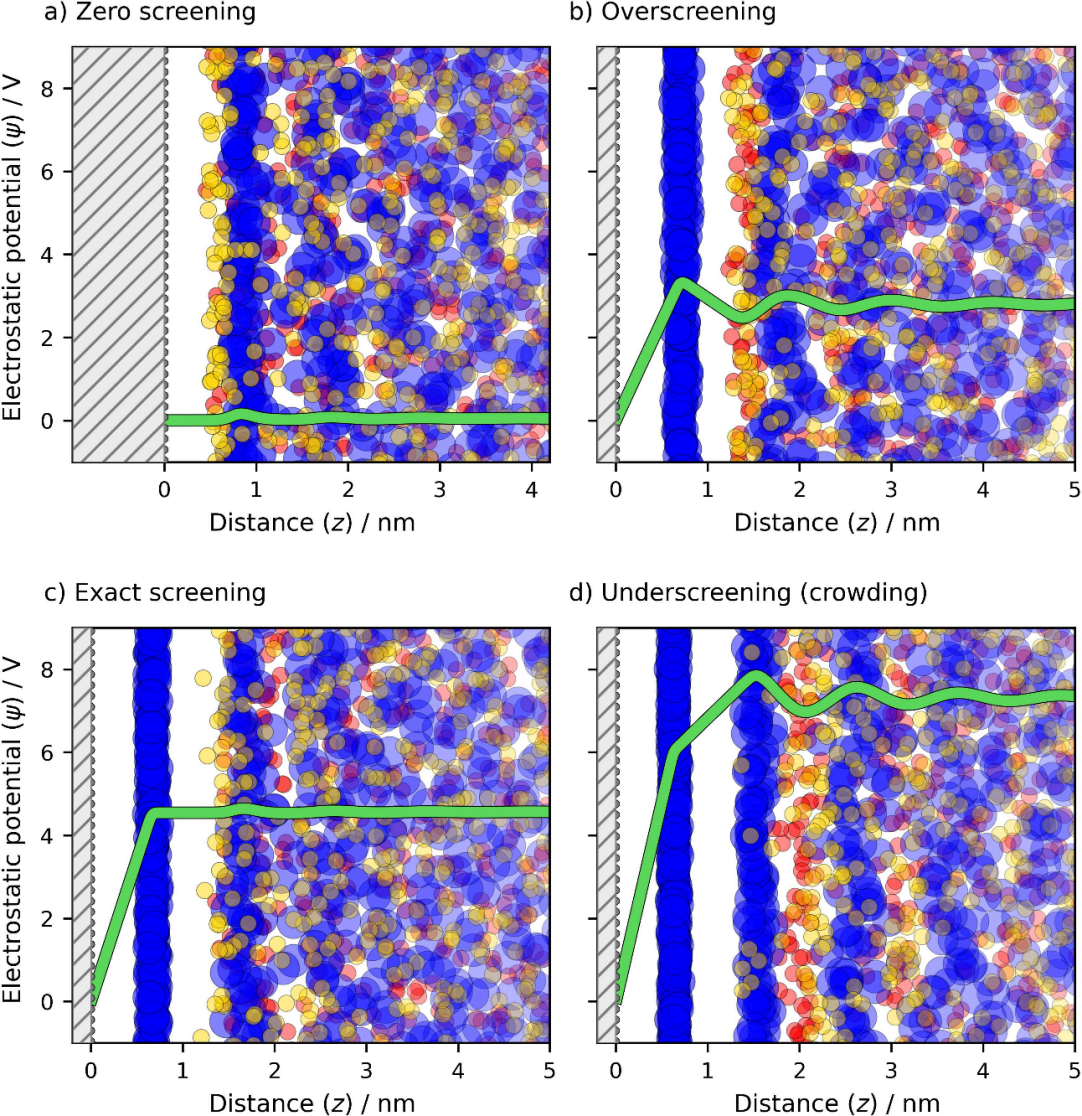
Schematic representation of an ionic liquid–electrode interface at variable surface charge densities (0, 12, 17, and 24 μC cm^–2^) that is screened by shown counter- and co-ions. These snapshots derived from coarse-grained MD simulations of LAr^–^ (blue Large Anions) and SCr^+^ (red Small Cations) with STr tails (yellow Small Tails) from refs 33 and 34 . The shift on the *x*-axis aligns EDL structures in (a) and (c) to show similarities in the potential profile and ions’ distribution in the bulk region where the surface charge is completely screened.

Hereafter, the term *potential* refers to the electrostatic potential drop, φ, across the EDL, relative to the potential of zero charge (PZC). The PZC was evaluated by averaging the potential drops from the electrodes to the bulk at zero surface charge in two-electrode simulations. The spatially resolved electrostatic potential obtained from simulations remains explicitly referred to as the *electrostatic potential*, ψ, like in Figure 2.

To distinguish between overscreening and crowding, it is convenient to refer to the effective charge planes for the electrode, contact layer, and the rest of the EDL as series-connected capacitors, like in the bilayer model.^15^ The core parameters of the bilayer model are the surface charge density (σ); the contact layer charge density (θ); the excess of the contact layer charge density, λ (λ = – θ – σ); the distance *l* between the surface and the contact layer charge planes; the distance δ between the contact layer and the charge plane that represents rest of the EDL and compensates λ; and the potential φ = (*l*σ – *δλ*)/*ε*_0_ *ε*_∞_, where *ε*_0_ is the permittivity of vacuum and *ε*_∞_ is the high-frequency dielectric constant. Using these definitions, we can phenomenologically describe the characteristic charging states, regimes, and mechanisms observed in MD simulations, such as those that give rise to the *C*_d_(φ) dependence in Figure 1.

Figure 2 illustrates the main screening regimes—overscreening and underscreening (crowding)—as well as the interfacial structures that emerge at two characteristic potentials: the PZC and the Potential of Monolayer Charge (PMC).^7,23^ The PMC, denoted below as φ_M_, naturally suits the relative potential scale, as in Figure 1b.

At the PZC (Figure 2a), the surface charge density is zero (σ = 0), and the electrostatic potential profile is flat, indicating the absence of ion layering. At higher electrode polarization than the PZC (Figure 2b), overscreening occurs through adsorption of counterions and swapping between anions and cations in the contact layer.^35^ This leads to the formation of distinct counter- and co-ion layers near the surface, i.e., the ion layering and overscreening.

At saturation, when ∂λ/∂σ = 0,^15,36^ the contact layer holds the maximum number density of co-ions. Accordingly, between the saturation state and the PMC, the charging process is governed by the desorption of co-ions into the bulk, as shown below. This results in the formation of the compact monolayer at the interface. At PMC (Figure 2c), φ_M_ ≈ *lσ*_M_/*ε*_0_ *ε*_∞_ and the electrostatic potential profile turns flat, indicating the disappearance of ion layering beyond the monolayer. Thus, both PZC and PMC correspond to exact surface charge screening.

At higher electrode polarization than the PMC, the EDL enters the *crowding* regime (Figure 2d), where the charging mechanism reverts to counterion adsorption. Notably, in terms of surface charge screening, there is no difference between removing a co-ion (− ⇌ + ) and adding a counterion (+ ⇌ – ) to the EDL beyond the contact layer. For this reason, upon saturation in both overscreening and crowding regimes, the capacitance–potential dependence follows a power law, which we discuss below from both theoretical and computational perspectives.

**Capacitance decay in the crowding regime**, in theory, follows the inverse-power-decay. In Section SI2, we remind the logic of deriving such dependence, which is finally expressed as

1 $$C_{d}=\frac{1}{2}K_{M}{\left(\frac{\varphi }{\varphi_{M}}\right)}^{-1/2}=C_{0}\sqrt{\frac{RT}{F}\cdot \frac{1}{2\gamma \left|\varphi \right|}}$$

where *K*_M_ = σ_M_/φ_M_ = *ε*_0_ *ε*_∞_/*l* is the monolayer integral capacitance. The second expression is written in terms of the Debye length, *l*_D_, and the Debye capacitance, *C*_0_ = *ε*_0_ *ε*_∞_/*l*_D_. It also introduces the ratio γ, defined as the average ion concentration *c* relative to the maximum local ion concentration *c*_max_, γ = *c*/*c*_max_. This expression is derived from mean-field theory under specific assumptions,^1^ as discussed in Section SI2.

**Capacitance decay in the overscreening regime** can be derived using the bilayer model as shown in Section SI3 and expressed as

2 $$C_{d}=\left[\frac{1}{2}K_{M}{\left(\frac{\varphi }{\varphi_{M}}\right)}^{-1/2}\right]\sqrt{\frac{{\left(\theta +\lambda \right)}^{2}}{\left(\theta +3\lambda \right)\theta }}\left\{1+f\left(\varphi \right)\right\}$$

where the term in square brackets is the square-root dependence and the term in figure brackets accounts for θ and λ dependence on φ. At high absolute potentials, when |θ| ≫ |λ| and *f*(φ) → 0, the root term is close to 1, and the capacitance can be approximated by an inverse-square-root dependence. Thus, above the saturation point, the power-law is valid for both overscreening and crowding regimes (eqs 1 and 2). In other words, the square-root dependence does not necessarily imply crowding, as was repeatedly stated in the past,^14,16–32^ but it can also indicate overscreening in the saturated regime. Therefore, the key message is that the power-law (*C*_d_ ∼ φ^*x*^) is characteristic of both overscreening and crowding regimes.

It is worth noting that continuum theories incorporating both steric constraints and ionic correlations, such as in refs 4 and 37 , provide a unified description of overscreening and crowding within a correlation-corrected mean-field framework. It predicts interfacial properties and their potential dependence. However, at moderate potentials, this unification becomes incomplete, as screening is increasingly governed by correlation-driven ionic clustering and network formation, which are not resolved within a mean-field description.^38^ This limitation motivates a structure-resolved perspective. In this work, we relate overscreening and crowding to screening mechanisms: In structure-resolved molecular dynamics simulations, overscreening and crowding are distinct regimes, defined directly from the mode of surface charge compensation by ions. From this viewpoint, the central result is that both regimes lead to the same asymptotic power-law decay of the differential capacitance, which explains why MFT performs (apparently) well in a wide potential range.

**Capacitance decay in MD simulations**, shown in Figure 1, follows the power-law in both overscreening and crowding regimes. We highlight the region in yellow that, in similar studies, was repeatedly misinterpreted as crowding—yet in fact belongs to the overscreening regime and follows a power-law.^14,16–32^ In the case of simulations, one can (re)­analyze the results to identify the PMC as described in Section SI6 or in refs 39 and 40 . For instance, a more careful analysis of MD simulations by Katakura et al. shows that the phenomena declared to occur in the crowding regime actually occur in the overscreening regime.^27,28,41^ Similar MD simulations of the same IL on Au­(*hkl*), made by different groups, show essentially the same results, yet lead to a different discussion;^14,42,43^ here the use of PMC as the reference potential along with the position of the surface charge plane and comparison of the σ_M_ values can lead to an objective and coherent picture. These two examples of “all’s well that ends well” highlight the benefit of referencing the PMC. However, we have previously concluded that in past experiments the PMC has never been reached,^40^ while in many past simulations, the PMC also remained outside the studied potential range. In those simulations, where the PMC was passed, a rather smooth transition between overscreening to crowding was observed on the *C*_d_(φ) dependence.^12,27,41,44^ Therefore, it is not enough to analyze *C*_d_(φ) on a log–log scale, but rather it is necessary to carefully examine the number densities with meaningful boundaries for ionic layers and relative to the PMC. All the more surprising, in Figure 1b, a breakpoint does appear at the PMC. The dependence above the PMC follows the inverse-square-root dependence above φ/φ_M_, which is evident in a slope of approximately – 1/2. Herewith, the other two slopes are less negative and follow a more general power-law:^41^

3 $$C_{d}\approx \alpha K_{M}{\left(\frac{\varphi }{\varphi_{M}}\right)}^{\alpha -1}$$

where α can be attributed to the packing geometry, as described in Section SI7, and the parameters *K*_M_ and φ_M_ for the state of exact surface charge screening by the monolayer of counterions, at the PMC, can be unambiguously determined via several literature methods.^39,40^ We now report a new approach to this task, and below we turn our attention to a more important aspect of the MD trajectory analysis – a more rigorous definition of the contact layer boundary.

**Analysis of MD simulations** is well-suited to study overscreening and crowding. However, analysis of MD trajectories requires attention to the boundaries of the ionic layering, because without a clear definition of boundaries “nothing will come of nothing”. Most previous MD studies have set a fixed boundary for the contact layer, approximately equal to the diameter of the counterion.^13,27,28^ For our coarse-grained IL model, this is ∼1 nm, as indicated with a vertical dotted line in Figure 3 representing the dependence of the number density of LAr^–^ and SCr^+^ ions on potential and distance from the electrode. As shown, within this boundary, the contact layer contains a mixture of co- and counterions. First, determining whether an ion is actually in contact with the surface is based on an arbitrary choice of the contact distance. This shows again that first impressions may be misleading, and the truth will only come out after close inspection. Second, even the distance between the surface and the nearest ions shows a wide distribution and depends on the potential.

**Figure 3:**
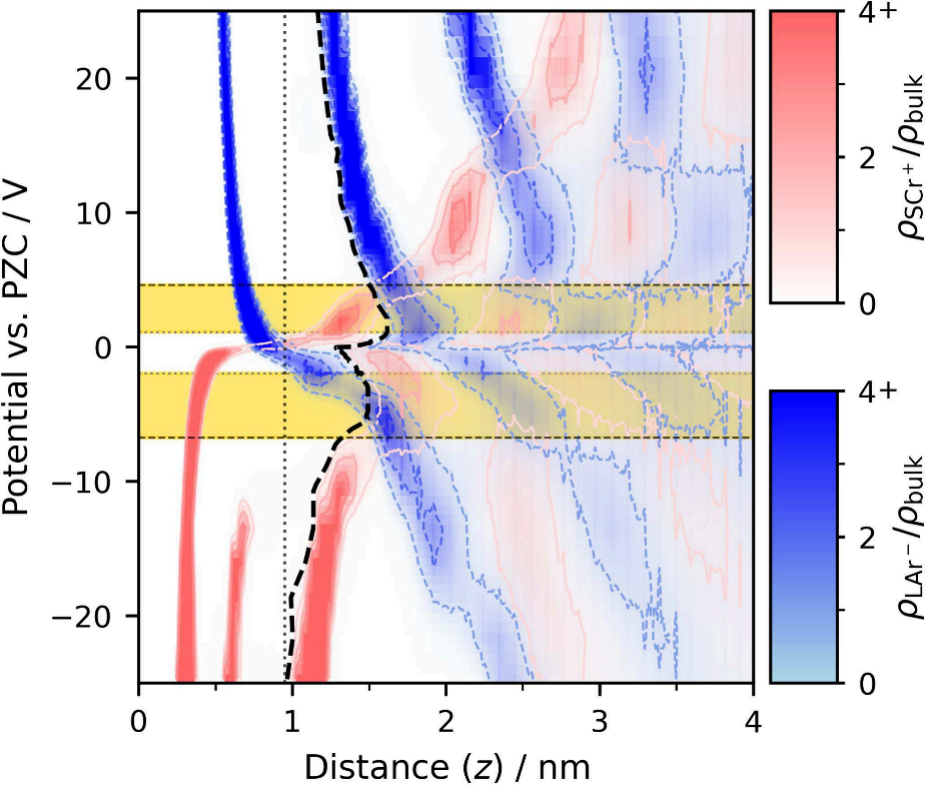
Dependence of the number density of ions on potential and distance from the electrode, with ρ_bulk_ = 0.65 nm^–3^. Data are from coarse-grained MD simulations of large anions (LAr^–^) and small cations (SCr^+^) with small tails (STr). Density levels of 1, 2, 3, and 4 relative to ρ_bulk_ are contoured to guide the eye.

To remove the associated ambiguity, we redefine the contact layer as the interfacial domain preceding the onset of the second counterion layer, as indicated by the bold dashed line in Figure 3. Here, the positions of counterion layers are identified directly from the peaks in the corresponding counterion number density profiles, which are well resolved at all studied potentials. The role of the contact layer definition is not to distinguish individual counterion layers, but to determine whether co-ions participate in screening within the interfacial region.

In practice, the first counterion layer and the first co-ion layer cannot be cleanly separated by a fixed geometric criterion, because their spatial distributions overlap and depend on local packing and ion reorientation. For this reason, we treat them jointly as a single contact layer. Within this framework, the presence of a finite co-ion density between the first two counterion layers provides the characteristic structural signature of the overscreening regime, whereas its absence indicates the crowding regime.

Operationally, we first introduce a virtual, smoothly varying boundary that excludes the counterion number density associated with the first-layer cluster. This boundary is positioned approximately at the midpoint of the dominant peaks in the counterion number density profiles (Figure SI1) and naturally accounts for the potential-dependent reorientation of SCr^+^–STr ions, as discussed in Section SI5. At a given potential, the onset of the second counterion layer is then identified as the first position farther from the electrode surface than this boundary at which the counterion number density reaches its bulk value of 0.65 nm^–3^. This criterion defines the outer limit of the interfacial region in which co-ions may exist. In the crowding regime, no co-ion layer is detected within this region, consistent with the complete exclusion of co-ions from the contact layer.

It is worth noting that this flexible boundary is a purely structural definition, based on the spatial packing of ions at the interface. This perspective is conceptually different from approaches based on energetic criteria and thermodynamic partitioning. Such as involving the Bjerrum-type ionic association or counterion condensation, as in descriptions of charged colloids and surfaces.^4,45^ Most importantly, this definition captures the strong attraction between anions and cations, which hold them together within the contact layer even when counterions are attracted, and co-ions are repelled by the charged surface.

The difference in the analysis using fixed and flexible boundaries is evident in Figure 4, which shows the number densities of co- and counterions in the contact layer. Markers denote values obtained using the flexible boundary, while the dashed line corresponds to results from the fixed boundary. The divergence is seen closer to the PZC. As observed in the literature, using a fixed boundary, one can conclude that co-ions are completely repelled from the contact layer at relatively low absolute potentials,^12,13,27,28^ such as 1 V in Figure 4. However, using the flexible boundary, one can see that co-ions are just losing contact with the surface (Figure 3), yet they remain very close to the surface. In Figure 4, this is evident from a constant value for the number density of co-ions within a wide potential window from – 2 to 2 V. Thus, we dare to conclude that in real experiments, the contact layer is almost always a mixture of both anions and cations, despite some layering, which mostly happens via swapping the surface positions of co-ions by counterions.

**Figure 4:**
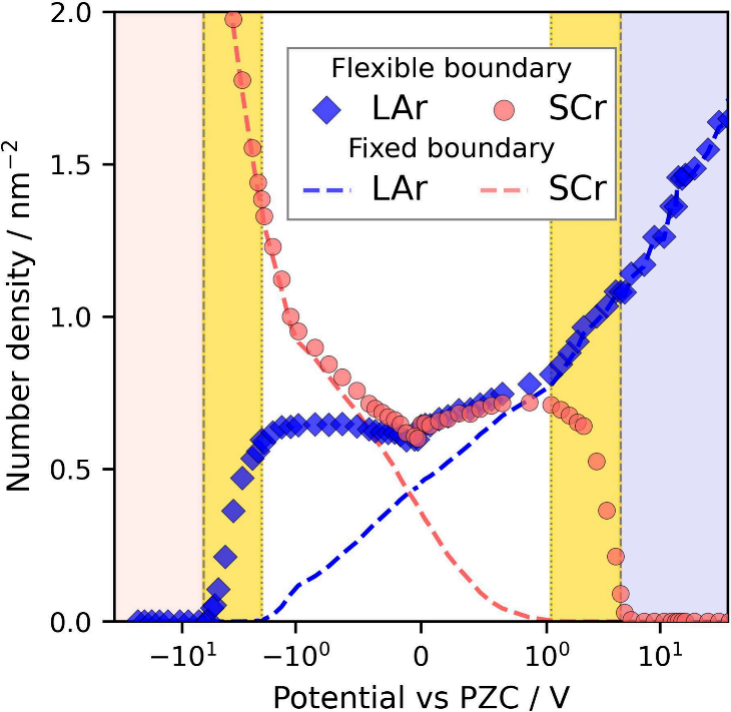
Number density of LAr^–^ and SCr^+^ ions within the fixed and flexible boundaries. The vertical dashed and dotted lines mark the PMC determined by charge excess analysis (see Section SI6) and the potential of co-ion depletion within the fixed boundary, respectively. The pale red and blue regions mark corresponding crowding regimes, whereas the yellow region highlights where overscreening in the saturation regime has been commonly misinterpreted as crowding.^14,16–32^

Let us pay attention to the potential regions highlighted with yellow in Figure 4 and 1. This is where interpretations of the MD data diverge across studies. Some interpret the absence of co-ions right at the surface as crowding.^14,20,27,28,32^ We demonstrate that this is misleading, as it falls within the wide potential range, particularly evident in Figure 1, which corresponds to overscreening in the saturated EDL. From Figure 4 it is evident that within the corresponding potential range, the desorption of the co-ions from the contact layer occurs at a pace comparable to the counterions adsorption, as indicated by their slopes. This is consistent with the above-mentioned phenomenological description of charging states, regimes, and mechanisms, as discussed earlier, as shown in Figure 2.

In this context, several published MD studies already provide high-quality, structure-resolved data suitable for the present analysis. In particular, the data set reported in ref 46 offers well-resolved ion density profiles over a broad potential range. Application of the present contact layer framework to such data may therefore yield additional insight into the role of co-ions and the distinction between overscreening and crowding, without requiring new simulations.

In conclusion, we show that the widely observed power-law decay of differential capacitance with increasing absolute potential does not necessitate the concept of counterion crowding. Consequently, the common interpretation that inverse-square-root dependence uniquely signals crowding must be reconsidered in favor of a broader view that includes overscreening at saturation.

Although much of the existing literature associates this capacitance decay with crowding,^14,16–32^ we emphasize that the overscreening mechanism alone is sufficient to produce the same asymptotic behavior. In particular, our analysis reveals that the same power-law scaling naturally emerges from the structural constraints of the EDL. Thus, with the presented power-law derivation, contact layer definition and PMC evaluation, the matter is at last plain, and the “to crowd or not to crowd” question can be settled with direct evidence. We hope this will motivate a systematic reevaluation of the crowding hypothesis in IL research, at both computational and theoretical levels. We encourage the community to apply the presented approaches to both new simulations and carefully designed experiments that can directly probe contact layer saturation in overscreening and crowding regimes.

## Supplementary Material



## Notes

Molecular dynamics simulation details, definitions and derivations of power-law capacitance decay in overscreening and crowding regimes, complete number density profiles and exclusion boundary construction, ion reorientation analysis, PMC determination by charge excess, and influence of ion packing geometry on capacitance scaling (PDF)
