# Supplementary material for: Ionic Liquid–Electrode Interface at Saturation: To Crowd, or Not to Crowd?
Source: J Phys Chem Lett. 2026 Feb 16;17(8):2374–9. doi: 10.1021/acs.jpclett.5c03710 (PMC12951558; doi:10.1021/acs.jpclett.5c03710)
Supplement: Supplementary file 1 [file jz5c03710_si_001.pdf]

Supporting Information: Ionic liquid–electrode interface at saturation: To crowd, or not to crowd?

Ba Long Nguyen,[†] Eva Roos Nerut,[†] Aleksandr Beditski,[‡] Nadezda Kongi,[†] Vladislav Ivaništšev,[*,†,¶] and Iuliia V. Voroshylova[*,§]

†*Institute of Chemistry, University of Tartu, Ravila 14a, 50411 Tartu, Estonia*

‡*Institute of Mathematics, University of Tartu, Narva mnt 18, 51009 Tartu, Estonia*

¶*Department of Chemistry, University of Latvia, Jelgavas iela 1, LV-1004 Riga, Latvia*

§*LAQV-REQUIMTE, Department of Chemistry and Biochemistry, Faculty of Sciences, University of Porto, 4169-007 Porto, Portugal*

E-mail: vladislav.ivanistsev@lu.lv; voroshylova.iuliia@fc.up.pt

1. Simulations details

To distinguish between overscreening and crowding regimes, we performed Molecular Dynamics simulations of the TMr model, consisting of large anions, LAr^-, and small cations, SCr^+, bearing neutral tails, STr. Here, TM denotes the original "tailed model", and the subscript r indicates that all ions are represented as charged, purely repulsive Lennard-Jones spheres, following the modelling approach of Ref. S1. The underlying TM model and its force field parameters are described in the work of Capozza et al.[S2,S3] The choice of the model enables comparison of the results with previous Molecular Dynamics and Monte Carlo simulations of coarse-grained ionic liquid models.

All simulations were carried out with GROMACS 2016.4 software at a temperature of 400 K and using the NaRIBaS scripting framework.[S4]

The general setup of the simulation box consisted of the following steps: The simulated bulk density of the IL was established with the 2017 TMr ion pairs in a cubic box after 15 ns NPT run; Two layers of graphite in hexagonal structure with dimensions in x and y of approximately 11.5 nm by 12 nm were generated with the Atomic Simulation Environment;[S5] The TMr ion pairs were randomly placed in a simulation box between graphite plates with the help of the Packmol package,[S6] taking into account the simulated bulk density of the IL. The distance between the electrodes in the final cell was approximately 23.5 nm.

The initial energy minimization, pre-equilibration (with a time step of 0.5 fs), and equilibration were run in the same way, as described in our previous work.[S7] The electrode polarization for 4 ns was performed as follows: an electric field was applied in the z direction, perpendicular to the electrodes, within the simulation box. The properties were sampled during a 20 ns long production run in the NVT ensemble. Surface charge density (σ) values of 0, 0.25, 0.5, 1, 1.5, 2, 2.5, 3, 3.5, 4, 5, 6, 7, 8, 9, 10, 12, 14, 16, 17, 18, 20, 24, 28, 32, 36, 38, 40, 42, 44, 50, 60, 70, 80, 90, 100, 110, 120 μC cm^{-2} and their opposite were applied to the two electrodes by distributing the total charge across their 572 constituent atoms.

The Verlet leapfrog algorithm was used to integrate the equations of motion for all simulations, with a time step of 10 fs.[S8] Periodic boundary conditions were applied only in x and y directions, considering the studied systems' slab-like geometry. The temperature was maintained at a constant value using a coupling constant of 1.0 ps for the velocity rescaling thermostat.[S9] All the cut-off distances, including short-range non-bonded Coulomb, Lennard-Jones interactions, and short-range neighbor list, were set to 5.4 nm. The 3dc Ewald geometry was employed,[S10] i.e., the reciprocal sum for Ewald sum evaluation was performed in 3D, but a force and potential correction were applied in the z dimension to produce a pseudo-2D summation. To correct the Coulomb interactions beyond the cut-off, the particle

mesh Ewald method[S11] with an interpolation order of 6 and the spacing of the grid points in the reciprocal space of 0.2 nm was used. Constraints on all bond lengths were enforced with the LINCS algorithm.[S12] The trajectories were written every 5 ps and then analyzed using GROMACS' in-built tools and in-house codes.

2. Power-law derivation for the crowding regime

In the crowding state, we can assume that: (1) only counter-ions determine the potential, (2) maximum counter-ion charge concentration (c_{max}) is constant. In this case, the effective position of the counter-ion charge density plane equals L while the EDL geometric width is $2L$. From $\sigma = 2qc_{\mathrm{max}}L$ and $\varphi = \sigma L/(\varepsilon_0\varepsilon_\infty)$ we get:

$$L = \sqrt{\frac{1}{2}\frac{\varepsilon_0\varepsilon_\infty\varphi}{qc_{\mathrm{max}}}} \tag{1}$$

Substituting that into the σ expression and taking the derivative gives:

$$C = \frac{\partial\sigma}{\partial\varphi} = \frac{1}{2}\sqrt{qc_{\mathrm{max}}\frac{2\varepsilon_0\varepsilon_\infty}{\varphi}} \tag{2}$$

Combining $\varphi_{\mathrm{M}} = 2l^2qc_{\mathrm{max}}/(\varepsilon_0\varepsilon_\infty)$ gives:

$$C = \frac{1}{2}K_{\mathrm{M}}\left(\frac{\varphi}{\varphi_{\mathrm{M}}}\right)^{-1/2} \tag{3}$$

where $K_{\mathrm{M}} = \sigma_{\mathrm{M}}/\varphi_{\mathrm{M}} = \varepsilon_0\varepsilon_\infty/l$ is the contact layer integral capacitance.

Alternatively, capacitance can be expressed using Debye length, $l_{\mathrm{D}} = \sqrt{\frac{RT}{F}\frac{\varepsilon_0\varepsilon_\infty}{q\gamma c_{\mathrm{max}}}}$, and Debye capacitance, $C_0 = \varepsilon_0\varepsilon_\infty/l_{\mathrm{D}}$, with the ratio between the average concentration of ions to the maximal possible local concentration of ions, $\gamma = c/c_{\mathrm{max}}$:

$$C = C_0\sqrt{\frac{RT}{F}\cdot\frac{1}{2\gamma|\varphi|}} \tag{4}$$

That is exactly the same expression as in the mean-field theory (MFT) at high $|\varphi|$ values.[S13] However, the derivation above explicitly accounts for the closest approach distance, whereas the MFT theory does not.[S13] The agreement appears when the correction for the surface–contact layer potential drop becomes negligible at high absolute potentials:

$$\frac{1}{C} = \frac{1}{C_\mathrm{c}} + \frac{1}{C_0\sqrt{\frac{RT}{F}\cdot\frac{1}{2\gamma\varphi}}} \to C = C_0\sqrt{\frac{RT}{F}\cdot\frac{1}{2\gamma\varphi}} \tag{5}$$

Let us note that in a similar derivation in Ref. S13, the differential capacitance was confused with the integral one, leading to a difference of $\sqrt{2}$ between formula 3 and the MFT theory. In fact, there is no difference.

3. Power-law derivation for the overscreening regime

Capacitance decay in the overscreening regime can be derived using the bilayer model.[S14]

To ease the algebra, let $\delta = 2l$ and the average charge densities within the first and second layers be given by the concentration of counter- and co-ions $q_\mp c_\mp$ and $q_\pm c_\pm$. Then the potential drop equals:

$$\varphi = [-l\theta - (l+\delta)\lambda]/\varepsilon_0\varepsilon_\infty = [2l^2 q_\mp c_\mp - 12l^2 q_\pm c_\pm]/\varepsilon_0\varepsilon_\infty \tag{6}$$

From these we get expression for l in terms of $\varphi_\mathrm{M} = -\theta_\mathrm{M} l/\varepsilon_0\varepsilon_\infty$ and $K_\mathrm{M} = \varepsilon_0\varepsilon_\infty/l$:

$$l = \sqrt{\frac{\varepsilon_0\varepsilon_\infty\varphi}{2q_\mp c_\mp - 12q_\pm c_\pm}} = K_\mathrm{M}\sqrt{\frac{\varphi\varphi_\mathrm{M}}{(2q_\mp c_\mp - 12q_\pm c_\pm)q_\mp c_\mp}} \tag{7}$$

Substituting l into the $\sigma = -(\theta + \lambda) = 2lq_{\pm}c_{\mp} - 4lq_{\pm}c_{\pm}$ and taking derivative gives:

$$C = \left[\frac{1}{2}K_{\mathrm{M}}\left(\frac{\varphi}{\varphi_{\mathrm{M}}}\right)^{-\frac{1}{2}}\right]\sqrt{\frac{(\theta+\lambda)^2}{(\theta+3\lambda)\theta}}\Big\{1 + f(\varphi)\Big\} \tag{8}$$

where the term in square brackets is the square-root dependence and the term in figure brackets accounts for θ and λ dependence on φ. At high absolute potentials, when $|\theta| \gg |\lambda|$ and $f(\varphi) \to 0$, the root term is close to 1, and the capacitance can be approximated by an inverse-square dependence.

4. Complete number density profiles

Figure S1 shows the complete number density dependence on potential and distance from the electrode with an exclusion boundary (dashed curves) for the flexible boundary detection.

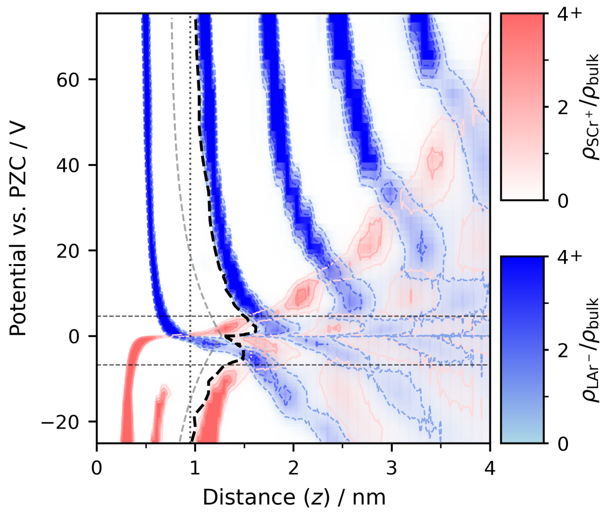

Figure S1: Dependence of the cubic number density of ions on potential and distance from the electrode, with $\rho_{\mathrm{bulk}} = 0.65\ \mathrm{nm}^{-3}$. Data are from coarse-grained MD simulations of LAr^{-} (large anions) and SCr^{+} (small cations) ions with STr (small tails). Density levels of 1, 2, 3, and 4 relative to ρ_{bulk} are contoured to guide the eye.

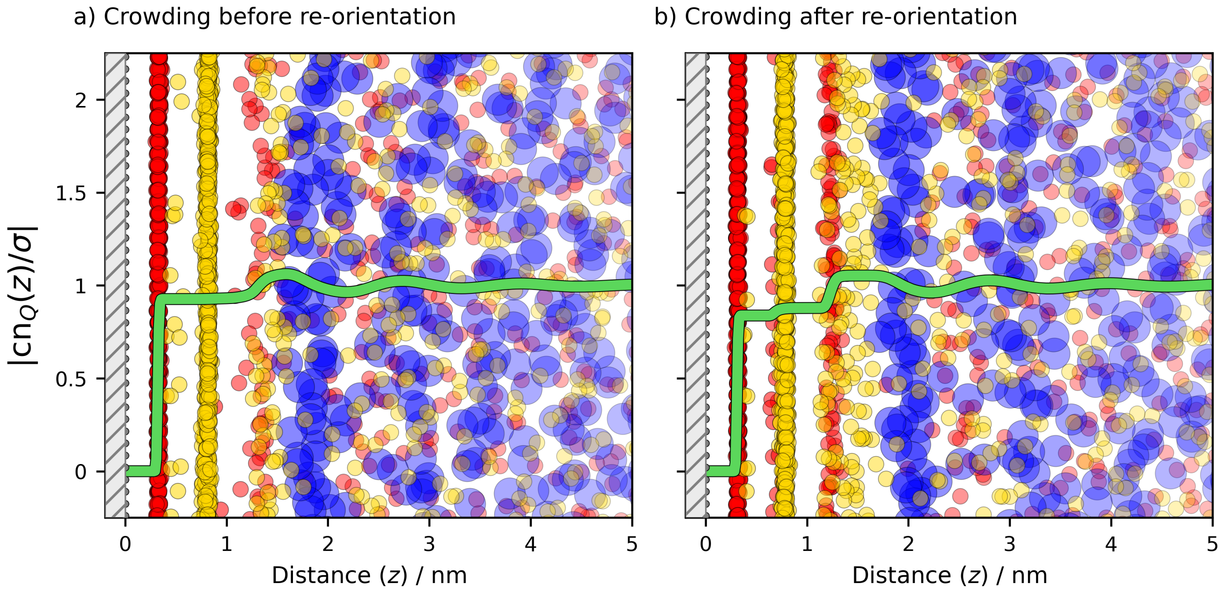

Figure S2: Schematic representation of an ionic liquid–electrode interface and normalized cumulative charge density profile dependence on the distance from the electrode at surface charge densities of 70 (a) and 80 $\mu C\,cm^{-2}$ (b). These snapshots derived from coarse-grained MD simulations of LAr^- (blue Large Anions) and SCr^+ (red Small Cations) with STr tails (yellow Small Tails) from Refs. S2,S3.

5. Reorientation of SCr^+–STr ions in the crowding regime

A careful reader may notice a seeming inconsistency in our definition of the contact layer. At potentials more negative than −12 V, the flexible boundary appears to miss a second SCr^+ peak. However, this feature does not correspond to a genuine second counter-ion cluster in the crowding regime. Instead, it arises from the reorientation of SCr^+–STr ions within the first-layer cluster.

Figure S2 clearly illustrates the structural change before and after reorientation. The position of the true second counter-ion layer remains essentially unchanged. The additional peak observed in the number density profiles originates from cation reorientation rather than from the formation of a new layer. This interpretation is further supported by the normalized cumulative surface charge density shown in Figure S2b, which exceeds unity only at the true second counter-ion layer located at approximately 1.2 nm.

6. PMC determination by charge excess analysis

Figure S3 shows the charge density gaps between the electrode surface and the contact layer – by definition, the charge excess (λ) for potentials below the PMC – and the ion density within the contact layer. Points denote data obtained using the dynamic boundary, while the dashed line corresponds to results from the static boundary.

From the charge density gap analysis, the PMCs of LAr^- and SCr^+ are readily determined as the potentials of zero charge excess: 4.6 V and −6.8 V, respectively. These values appear robust, with strong agreement between results obtained from the two boundary definitions. Namely, the charge density in the first layer can be straightforwardly extracted in this case.

Furthermore, the obtained PMCs are consistent with the contact layer ion densities given by the dynamic boundary. At these potentials, co-ions are virtually absent from the contact layer. By contrast, the fixed boundary yields co-ion depletion potentials that lie far beyond these values (1.1 V and −2.0 V, respectively), thereby underestimating the ion density in the region between the given border and the second counter-ion layer.

While the charge excess appears to be a meaningful criterion for identifying PMCs, we anticipate that, in modern MD simulations where charge profiles are significantly more intricate, the contact-region density analysis with dynamic boundaries will remain both applicable and straightforward for estimating PMCs with slight deviations. For coarse-grained models, this advantage is particularly evident, as the charge can be computed directly from the number of ion "balls".

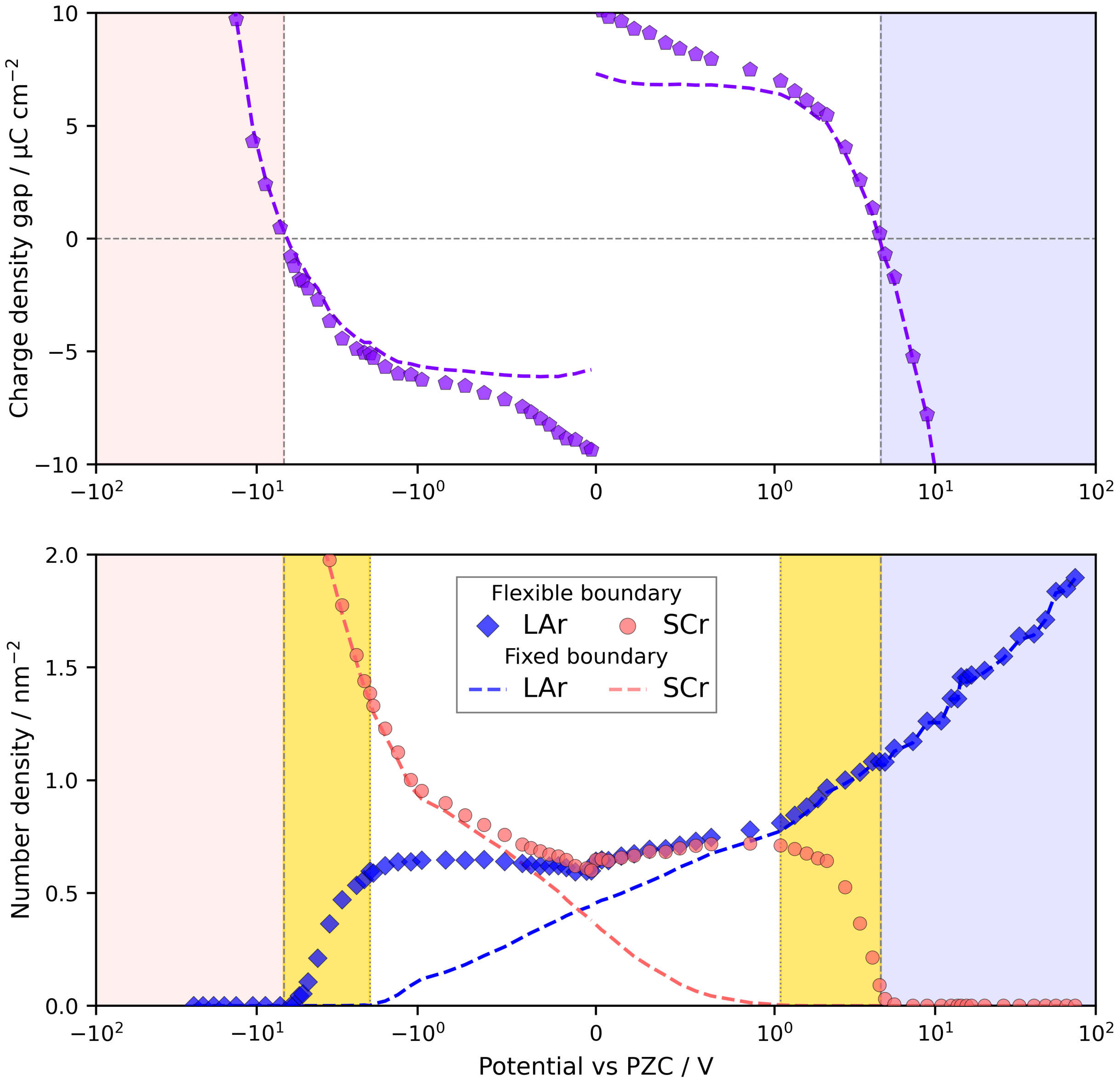

Figure S3: Top: Difference between the charge densities of the electrode surface and the counter-ions in the contact layer. Bottom: Number density of LAr^- and SCr^+ ions within the contact-region. The vertical dashed and dotted lines indicate the PMC determined by charge excess analysis and the potential of co-ion depletion within the contact layer, respectively, as defined by the fixed boundary. The pale red and blue regions mark corresponding crowding regimes, whereas the yellow region highlights where overscreening in the saturation regime has been commonly misinterpreted as crowding.

7. Power-law dependence on ion packing

The above-presented derivation of the inverse-square-root dependence for the crowding regime implies close packing of ions, which, in the original article, is referred to as occupation of lattice positions.[S13] Below, we examine the dependence of the power-law on the ion packing for primitive cubic, hexagonal close-packed, and face-centered cubic structures. This indicates that the deviation from the ideal inverse-square-root relationship is partly due to ion packing.

Primitive cubic structure

The dependence of the surface charge on the interlayer distance:

$$\sigma(l) = -qc_{\max}nl$$

The dependence of the potential on the surface charge:

$$\varphi(L) = \frac{\sigma(l)}{\epsilon}L$$

Distance l between layers in the case of a primitive cubic structure is just $2r$:

$$l = 2r$$

Hence, the surface charge is:

$$\sigma(n) = -qc_{\max}nl = -2rqc_{\max}n$$

The effective surface of the charge is located in the center of the charges at:

$$L = \frac{2r + (n-1)l}{2} = rn$$

and hence the dependence of the potential on n is:

$$\varphi(n) = -\frac{2r^2 q c_{\text{max}} n^2}{\epsilon}$$

$$\varphi(1) = -\frac{2r^2 q c_{\text{max}}}{\epsilon}$$

Expressing potential as a dimensionless quantity:

$$\varphi_0(n) = \frac{\varphi(n)}{\varphi(1)} = n^2$$

The value of the surface charge at $n = 1$:

$$\sigma(1) = -2rqc_{\text{max}}$$

Expressing surface charge as a dimensionless quantity:

$$\sigma_0(n) = \frac{\sigma(n)}{\sigma(1)} = n$$

Hexagonal close-packed structure

Distance l between layers in the case of a hexagonal closed packing structure is the height of a tetrahedron :

$$l = \frac{2r\sqrt{6}}{3}$$

Hence, the surface charge is:

$$\sigma(n) = -qcnl = \sigma(n) = -\frac{2\sqrt{6}rqc_{\text{max}}n}{3}$$

The effective surface of the charge is located in the center of the charges at:

$$L = \frac{2r + (n-1)l}{2} = \frac{r(n\sqrt{6} + (3-\sqrt{6}))}{3}$$

and hence the dependence of the potential on n is:

$$\varphi(n) = -\frac{1}{\epsilon}\frac{2rqc_{\text{max}}n\sqrt{6}}{3}\frac{r(n\sqrt{6} + (3-\sqrt{6}))}{3} = -\frac{2r^2qc_{\text{max}}}{9\epsilon}\left(n^2\sqrt{6} + n(3-\sqrt{6})\right)$$

The value of potential at $n = 1$:

$$\varphi(1) = -\frac{2r^2qc_{\text{max}}}{9\epsilon}\left(\sqrt{6} + (3-\sqrt{6})\right) = -\frac{r^2qc_{\text{max}}}{9\epsilon}6$$

Expressing potential as a dimensionless quantity:

$$\varphi_0(n) = \frac{\varphi(n)}{\varphi(1)} = \frac{n^2\sqrt{6} + n(3-\sqrt{6})}{3}$$

The value of the surface charge at $n = 1$:

$$\sigma(1) = -\frac{2\sqrt{6}rqc_{\text{max}}}{3}$$

Expressing surface charge as a dimensionless quantity:

$$\sigma_0(n) = \frac{\sigma(n)}{\sigma(1)} = n$$

Face-centred cubic structure

Distance l between layers in the case of the fcc structure is:

$$l = r\sqrt{2}$$

Hence, the surface charge is:

$$\sigma(n) = -\sqrt{2} r q c_{\max} n$$

The effective surface of the charge is located in the center of the charges at:

$$L = \frac{2r + (n-1)l}{2} = \frac{2r + r(n-1)\sqrt{2}}{2} = \frac{r(2 + (n-1)\sqrt{2})}{2} = \frac{r(n\sqrt{2} + (2-\sqrt{2}))}{2}$$

and hence the dependence of the potential on n is:

$$\varphi(n) = -\frac{r q c_{\max} n \sqrt{2}}{\epsilon} \frac{r(n^2\sqrt{2} + (2-\sqrt{2}))}{2}$$

$$= -\frac{r^2 q c_{\max}}{2\epsilon}(n^2\sqrt{2} + (2-\sqrt{2}))$$

The value of potential at $n = 1$:

$$\varphi(1) = -\frac{r^2 q c_{\max}}{2\epsilon}(\sqrt{2} + (2-\sqrt{2})) = -2 \cdot \frac{r^2 q c_{\max}}{2\epsilon}$$

Expressing potential as a dimensionless quantity:

$$\varphi_0(n) = \frac{\varphi(n)}{\varphi(1)} = \frac{n^2\sqrt{2} + n(2-\sqrt{2})}{2}$$

The value of the surface charge at $n = 1$:

$$\sigma(1) = -\sqrt{2} r q c_{\text{max}}$$

Expressing surface charge as a dimensionless quantity:

$$\sigma_0(n) = \frac{\sigma(n)}{\sigma(1)} = n$$

Asymptotic analysis

We now express the capacitance in the crowding regime as a function of the dimensionless potential. In all three packing, the reduced surface charge is simply:

$$\sigma_0(n) \equiv \frac{\sigma(n)}{\sigma(1)} = n$$

while the dimensionless potential can be written in the generic quadratic form

$$\varphi_0(n) = an^2 + bn$$

with (a, b) determined by the packing geometry. By definition, the reduced differential capacitance is

$$\tilde{C}(n) \equiv \frac{d\sigma}{d\varphi} \Big/ \frac{\sigma(1)}{\varphi(1)} = \frac{d\sigma_0}{d\varphi_0} = \left(\frac{d\varphi_0}{dn}\right)^{-1} = \frac{1}{2an + b}$$

Inverting the quadratic relation $\varphi_0 = an^2 + bn$ gives:

$$n(\varphi_0) = \frac{-b + \sqrt{b^2 + 4a\varphi_0}}{2a}$$

so that

$$\tilde{C}(\varphi_0) = (2an(\varphi_0) + b)^{-1} = \frac{1}{\sqrt{b^2 + 4a\varphi_0}}$$

The coefficients a and b differ between primitive cubic, hexagonal close-packed, and face-centered cubic lattices. For the primitive cubic lattice, this gives a pure ${\varphi_0}^{-1/2}$ dependence.

Equivalently, the quadratic relation $\varphi_0(n) = an^2 + bn$ implies a local power–law between n and φ_0. Defining the instantaneous exponent as:

$$\alpha(\varphi_0) \equiv \frac{d \ln n}{d \ln \varphi_0} = \frac{\varphi_0}{n} \frac{dn}{d\varphi_0} = \frac{an + b}{2an + b}$$

We can write, in a narrow neighbourhood of a given φ_0:

$$n(\varphi_0) \sim \varphi_0^{\alpha(\varphi_0)}$$

Since $\sigma \propto n$, this yields a corresponding local scaling of the differential capacitance:

$$C_{\mathrm{d}}(\varphi_0) = \frac{d\sigma}{d\varphi_0} \sim \alpha(\varphi_0)\, \varphi_0^{\alpha(\varphi_0)-1}$$

This is equivalent to the generalized power-law given in the main article text:

$$C \approx \alpha K_{\mathrm{M}} \left(\frac{\varphi}{\varphi_{\mathrm{M}}}\right)^{\alpha-1} \tag{9}$$

In the strongly crowded regime ($n \gg 1$) we have $\alpha(\varphi_0) \to 1/2$ for all three packing, so that $C_{\mathrm{d}}(\varphi_0) \propto \varphi_0^{-1/2}$ independently of the specific lattice structure, while deviations at intermediate crowding reflect the packing–dependent variation of $\alpha(\varphi_0)$.

At the PMC, $n = 1$ and $\alpha = (a+b)/(2a+b)$, thus, it has a geometrical meaning and is defined by the packing of ions.

References

(S1) Kirchner, K.; Kirchner, T.; Ivaništšev, V.; Fedorov, M. Electrical Double Layer in Ionic Liquids: Structural Transitions from Multilayer to Monolayer Structure at the Interface. *Electrochimica Acta* **2013**, *110*, 762–771.

(S2) Capozza, R.; Benassi, A.; Vanossi, A.; Tosatti, E. Electrical Charging Effects on the Sliding Friction of a Model Nano-Confined Ionic Liquid. *The Journal of Chemical Physics* **2015**, *143*, 144703.

(S3) Capozza, R.; Vanossi, A.; Benassi, A.; Tosatti, E. Squeezout Phenomena and Boundary Layer Formation of a Model Ionic Liquid under Confinement and Charging. *The Journal of Chemical Physics* **2015**, *142*, 064707.

(S4) Roos Nerut, E.; Karu, K.; Voroshylova, I. V.; Kirchner, K.; Kirchner, T.; Fedorov, M. V.; Ivaništšev, V. B. NaRIBaS—A Scripting Framework for Computational Modeling of Nanomaterials and Room Temperature Ionic Liquids in Bulk and Slab. *Computation* **2018**, *6*, 57.

(S5) Larsen, A. H.; Mortensen, J. J.; Blomqvist, J.; Castelli, I. E.; Christensen, R.; Dulak, M.; Friis, J.; Groves, M. N.; Hammer, B.; Hargus, C.; Hermes, E. D.; Jen-

nings, P. C.; Jensen, P. B.; Kermode, J.; Kitchin, J. R.; Kolsbjerg, E. L.; Kubal, J.; Kaasbjerg, K.; Lysgaard, S.; Maronsson, J. B.; Maxson, T.; Olsen, T.; Pastewka, L.; Peterson, A.; Rostgaard, C.; Schiøtz, J.; Schütt, O.; Strange, M.; Thygesen, K. S.; Vegge, T.; Vilhelmsen, L.; Walter, M.; Zeng, Z.; Jacobsen, K. W. The Atomic Simulation Environment—a Python Library for Working with Atoms. *Journal of Physics: Condensed Matter* **2017**, *29*, 273002.

(S6) Martínez, L.; Andrade, R.; Birgin, E. G.; Martínez, J. M. PACKMOL: A Package for Building Initial Configurations for Molecular Dynamics Simulations. *Journal of Computational Chemistry* **2009**, *30*, 2157–2164.

(S7) Voroshylova, I. V.; Lembinen, M.; Ers, H.; Mišin, M.; Koverga, V. A.; Pereira, C. M.; Ivaništšev, V. B.; Cordeiro, M. N. D. S. On the Role of the Surface Charge Plane Position at Au(*hkl*)–$BMImPF_6$ Interfaces. *Electrochimica Acta* **2019**, *318*, 76–82.

(S8) Hockney, R. W.; Goel, S. P.; Eastwood, J. W. Quiet High-Resolution Computer Models of a Plasma. *Journal of Computational Physics* **1974**, *14*, 148–158.

(S9) Bussi, G.; Donadio, D.; Parrinello, M. Canonical Sampling through Velocity Rescaling. *The Journal of Chemical Physics* **2007**, *126*, 014101.

(S10) Yeh, I.-C.; Berkowitz, M. L. Ewald Summation for Systems with Slab Geometry. *The Journal of Chemical Physics* **1999**, *111*, 3155–3162.

(S11) Darden, T.; York, D.; Pedersen, L. Particle Mesh Ewald: An N·log(N) Method for Ewald Sums in Large Systems. *The Journal of Chemical Physics* **1993**, *98*, 10089–10092.

(S12) Hess, B.; Bekker, H.; Berendsen, H. J. C.; Fraaije, J. G. E. M. LINCS: A Linear Constraint Solver for Molecular Simulations. *Journal of Computational Chemistry* **1997**, *18*, 1463–1472.

(S13) Kornyshev, A. A. Double-Layer in Ionic Liquids: Paradigm Change? *Journal of Physical Chemistry B* **2007**, *111*, 5545–5557.

(S14) Ers, H.; Voroshylova, I. V.; Pikma, P.; Ivaništšev, V. B. Double Layer in Ionic Liquids: Temperature Effect and Bilayer Model. *Journal of Molecular Liquids* **2022**, *363*, 119747.
